# Molecular signatures of skeletal muscle insulin resistance: bringing personalised diabetes treatment a step closer

**DOI:** 10.1038/s41392-025-02412-7

**Published:** 2025-10-01

**Authors:** Giovanni Levate, Roland H. Stimson

**Affiliations:** University/ BHF Centre for Cardiovascular Science, University of Edinburgh, EH16 4TJ Edinburgh, UK

**Keywords:** Biological techniques, Physiology

In a recent article published in *Cell*, Kjaergaard et al. show that the skeletal muscle proteome and phosphoproteome are associated with whole-body insulin sensitivity, and identify selective insulin resistance (IR) signatures in individuals with and without type 2 diabetes (T2D).^1^ Their elegant work provides compelling evidence for more personalised therapeutic strategies to treat T2D.

T2D is a major public health burden, of which the cardinal features are insulin resistance and β-cell failure.^2^ In IR, most commonly induced by excess adiposity, insulin signalling becomes impaired in key target tissues, including skeletal muscle, liver, and adipose tissue. Skeletal muscle is responsible for substantial glucose uptake, particularly post-prandially during insulin stimulation. This tissue is a major site of early IR in individuals pre-disposed to develop T2D, in part due to reduced translocation of the insulin-sensitive glucose transporter 4 (GLUT-4) to the cell surface, leading to reduced glucose uptake and subsequent glycogen synthesis. Hyperinsulinaemia due to IR then drives de-novo lipogenesis and ectopic fat deposition in tissues such as the liver and skeletal muscle, further exacerbating IR.^3^ The aetiology of T2D is multifactorial, and IR in T2D patients is highly heterogeneous, due to differences in molecular pathways manifesting in differing degrees of insulin sensitivity or resistance in target organs. This, together with differences in the pathways leading to β-cell failure, contributes to variability in patients’ responses to pharmacological therapies. Therefore, personalised diagnostic tools, in addition to standard clinical markers such as blood glucose and HbA1c, could enhance the choice of therapy over a one-size-fits-all approach to T2D.

**Fig. 1:**
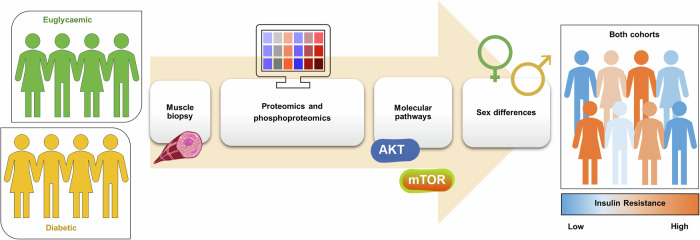
High-resolution mass spectrometry analysis was performed on skeletal muscle biopsies from euglycaemic individuals and patients with T2D, revealing the muscle proteome and phosphoproteome correlated with whole-body insulin sensitivity rather than diabetic status. Specific insulin resistance signatures were detected during insulin stimulation, with both Akt and mTOR pathways correlating with insulin sensitivity. Sex-specific differences in both proteome and phosphoproteome were also observed. Illustration uses icons by Servier Medical Art (https://smart.servier.com/), licensed under CC BY 4.0 (https://creativecommons.org/licenses/by/4.0/)

In this study, the authors sought to identify proteome-phenotype associations between different metabolic pathways and protein profiles with IR in human skeletal muscle. Individuals (comprising both sexes, females were peri- or post-menopausal to reduce confounders) with either normal glucose levels or T2D were recruited, 77 participants were used for the discovery cohort, with 46 used for a separate validation cohort, which is a considerable strength of this research. Each subject underwent two skeletal muscle biopsies (vastus lateralis) under fasting conditions, one prior to and one during an hyperinsulinaemic-euglycemic clamp. Proteomic and phosphoproteomic analyses were then performed using high-resolution mass spectrometry, enabling quantification of ~3000 proteins and 15,000 phospho-sites (Fig. 1).

The most striking finding of this study was that the fasting proteome and phosphoproteome were associated with whole-body IR, and not associated with diabetic status. In addition, specific components of insulin signalling were preserved in the skeletal muscle of some insulin-resistant T2D patients, revealing that certain pathways remain unaffected by IR. Insulin signalling transduction in peripheral organs relies on the activation of key kinases such as Akt and AMP-activated protein kinase (AMPK), and both enzymes were downregulated in T2D patients, as expected.^3,4^ In addition to confirming alterations in these classic pathways, IR was associated with disruption of mammalian target of rapamycin (mTOR) signalling, as the mTOR substrate sites AMPKα2 S377 and regulatory-associated protein of mTOR (RPTOR) S863 both correlated with insulin sensitivity. Interestingly, HbA1c and fasting glucose levels, measurements used to diagnose diabetes, did not correlate with the skeletal muscle proteomics or phosphoproteomics.

Pathway analysis revealed that proteins linked to fatty acid degradation, oxidative phosphorylation, and mitochondrial protein content positively correlated with insulin sensitivity, while Wnt signalling, and ubiquitin and lysosome proteolysis were directly associated with insulin resistance. Furthermore, the authors identified a novel phospho-site unique to humans, AMPKγ3 S65, that was highly associated with IR, along with its upstream kinase MAP kinase-activated protein kinase (MAPKAPK)-2, which is a downstream target of both p38 and c-Jun N-terminal Kinase (JNK).

Finally, the authors discovered distinct patterns of metabolic signalling between the two sexes. Proteins involved in glucose metabolism and oxidative phosphorylation were enriched in males, while those regulating lipid uptake and storage were enriched in females, in accordance with the literature.^5^ Most interestingly, sex was not a substantial determinant of the associations between the proteome/ phosphoproteome and insulin sensitivity, highlighting that the mechanisms of IR are conserved between both sexes.

A key strength of this work is the identification of novel proteins and signalling pathways that are dysregulated in IR. The identification of a sex-specific proteomic atlas is also a very powerful tool for future research. Validation of these molecular markers in larger, more diverse cohorts is needed to determine the applicability of these findings to other populations. Most excitingly, further research is needed to dissect causation from association, and crucially to identify candidates with therapeutic potential to improve insulin sensitivity in patients with substantial IR.

Very few current T2D treatments primarily target IR, with even those that do, such as thiazolidinediones out of favour for various reasons, including significant weight gain. As such, development of effective, safe insulin-sensitising agents would be a valuable addition to the field. However, the explosion in the use of incretin mimetics such as tirzepatide in this patient group, which reduces appetite and food intake, leading to substantial weight loss, targets excess adiposity, which is the primary cause of insulin resistance. The benefits of insulin-sensitising agents in this current landscape will need to be determined in due course.

This research also highlights that muscle and/or whole-body IR does not necessarily translate into the development of T2D, as proteomics could not discriminate between euglycaemic and T2D groups, and a ‘second hit’ is required with β-cell failure to develop the condition. Muscle biopsies for proteomics also represent an invasive, uncomfortable costly procedure, and circulating measures of IR, such as HOMA-IR, would be easier and cheaper to undertake clinically.

In conclusion, this exciting research highlights how current euglycemic therapeutic approaches overlook individual susceptibility and genetic predisposition to IR in T2D, and sets the stage for the use of precision medicine to improve treatment of T2D and potentially other metabolic diseases in the future.
